# Enterovirus 71-Induced Neurological Disorders in Young Gerbils, *Meriones unguiculatus*: Development and Application of a Neurological Disease Model

**DOI:** 10.1371/journal.pone.0051996

**Published:** 2012-12-21

**Authors:** Ping-Ping Yao, Lei Qian, Yong Xia, Fang Xu, Zhang-Nv Yang, Rong-Hui Xie, Xiao Li, Wei-Feng Liang, Xiao-Xiao Huang, Zhi-Yong Zhu, Han-Ping Zhu

**Affiliations:** 1 Key Lab of Vaccine against Hemorrhagic Fever with Renal Syndrome, Zhejiang Province Center for Disease Prevention and Control, Hangzhou, China; 2 Department of Pathology, First Municipal Hospital of Hangzhou, Hangzhou, Zhejiang Province, China; 3 Hangzhou Sixth People’s Hospital, Zhejiang, China; National University of Singapore, Singapore

## Abstract

A reliable disease model mimicking Enterovirus 71 (EV71) infection in humans is essential for understanding pathogenesis and for developing a safe and effective vaccine. Commonly used rodent models including mouse or rat models are not suitable for vaccine evaluation because the rodents are resistant to EV71 infection after they reach the age of 6 days. In this study, 21-day-old gerbils inoculated intraperitoneally (IP) with a non mouse-adapted EV71 strain developed neurological lesion-related signs including hind limb paralysis, slowness, ataxia and lethargy similar to those of central nervous system (CNS) infection of EV71 in humans. The infected gerbils eventually died of the neurological lesions and EV71 could be isolated from lung, liver, spleen, kidney, heart, spinal cord, brain cortex, brainstem and skeletal muscle. Significantly high virus replication was detected in spinal cord, brainstem and skeletal muscle by cellular analysis, real-time quantitative PCR (RT-PCR) and immunohistochemical staining. Histopathologic changes such as neuronal degeneration, neuronal loss and neuronophagia were observed in spinal cord, brain cortex, brainstem, and skeletal muscle along with necrotizing myositis and splenic atrophy. Gerbils that received two doses of inactive whole-virus vaccine showed no EV71-specific symptoms after challenged with EV71. In contrast, gerbils that received mock vaccination died of EV71-induced neuropathology after challenged with EV71. The result indicates that gerbils can serve as a reliable disease model for evaluating safety and efficacy of EV71 vaccine.

## Introduction

Enterovirus 71 (EV71) is a neurotropic virus belonging to the genus *Enterovirus* in the *Picornaviridae* family. It causes outbreaks of hand, foot and mouth disease (HFMD) in young children throughout the world with a significantly increased mortality in recent years, especially in the Asia-Pacific region [1], [2], [3], [4], [5]. While most EV71 infections result in mild diseases such as HFMD and herpangina, severe diseases such as aseptic meningitis, encephalitis, poliomyelitis-like paralysis, and pulmonary edema are also reported [6], [7], [8]. Fatal cases were mainly found in children under 3 years of age. Since the first case reported in California in 1969 [9], EV71 has caused several large-scale outbreaks worldwide and severe neurological diseases have been commonly diagnosed in young children [1], [10], [11], [12], [13]. In 2008, 488,955 HFMD cases were reported in China, and 126 children died of the infection. In this outbreak, EV71 was confirmed as the major pathogen [2]. The death was mainly due to EV71-induced severe neurologic complications, including extensive neuronal degeneration, CNS inflammation and pulmonary congestion with hemorrhage. Disease pathogenesis of the viral infection remains unclear, and currently there are no effective vaccines or therapeutic interventions available for EV71 infection [14]. Therefore, HFMD associated with EV71 infection is an important public health problem [15] and further understanding pathogenesis of the EV71 infection is needed to identify options for prevention and treatment of the disease.

The lack of a suitable disease model has been a major obstacle for understanding pathogenesis of EV71 infection. It has also hindered progress in developing effective vaccines and therapeutic approaches [16]. Experimental infections with EV71 have been reported in neonatal, 7-day-old, and 14-day-old mice [17], [18], [19]. Because the immune system in neonatal mice is premature and vaccination regimens take time, the models using newborn mice are not suitable for evaluating vaccine candidates. In this study, we used 21-day-old gerbils as an EV71 infection model and found that gerbils were susceptible to EV71 infection at this relatively older age. Furthermore, the EV71-infected gerbils showed CNS symptoms similar with patients. This animal model can be further developed as a useful disease model for understanding pathogenesis of EV71 infection, evaluating safety and efficacy of EV71 vaccine candidates and developing therapeutic interventions.

## Materials and Methods

### EV71 Virus Preparation

EV71 clinical isolate (strain 58301 genotype C4) was obtained from a twelve-month-old patient who suffered from mild HFMD in the Hangzhou Sixth People’s Hospital, Hangzhou China. A written informed consent was obtained from the parents of the patient. The study protocol was approved by the Hangzhou Sixth People’s Hospital Ethics Committee. Virus was grown in Vero cells. The titer for the virus stock was 1×10^8.0^ tissue culture infection dose (TCID_50_) determined by the standard method of assay in Vero cells, which were maintained in modified Eagle’s medium (MEM) containing 10% FBS [20].

### Animal Model

21-day-old gerbils were obtained from the Animal Center of Zhejiang Academy of Medical Sciences, Hangzhou, China. The animal care and use protocols were carried out according to the Regulations for the Administration of Affairs Concerning Experimental Animals of the People’s Republic of China and were approved by the Zhejiang Provincial Center for Disease Control and Prevention Institutional Animal Care and Use Committee. Seven groups (n = 7 or 8 per group) were inoculated IP with serially diluted EV71 (from 10^−1^ to 10^−7^). The animals were observed twice daily for clinical signs, weight gain or loss and mortality for 20 days. The 50% lethal dose (LD_50_) was determined as described by Reed and Muench [21]. Based on the LD_50_ study, another sixteen 21-day-old gerbils were divided into two groups, and were inoculated with 100×LD_50_ virus (about 1×10^5.0^TCID_50_). In the first group, 12 animals were sacrificed and virus titer measurements were made in selected tissues at different time points (3 h, 1 day, 3 days and 5 days). In the second group, 4 animals were subjected to histopathological examination and immunohistochemistry detection. Briefly, the gerbils were euthanized under anesthesia at the onset of clinical signs. After perfusion with isotonic saline containing EDTA, tissue samples were aseptically collected from heart, liver, spleen, lung, kidney, intestine, limb muscle, brain cortex, spinal cord and brainstem. The samples were homogenized in sterile PBS (10%, wt/vol), disrupted by freezing and thawing three times. Tissue suspensions were then obtained by centrifugation at 1000 g for 5 min. Virus titers in tissue supernatants and blood were measured in Vero cells as described previously [22] and displayed as log_10_ TCID_50_ per gram of tissue or per milliliter of blood. The control animals were inoculated with uninfected Vero cell lysate.

### Histopathology and Immunohistochemistry

Tissue samples from the gerbils inoculated with EV71 were fixed in 10% formalin for 48 h. The paraffin-embedded tissues were sliced and the sections were mounted on poly-L-lysine-coated slides. Sections were bisected. One part of the section was stained with hematoxylin and eosin or Nissl stain (CNS samples) for morphological examination and another part of the section was used for immunohistochemistry. Sections were dewaxed and incubated with 0.3% H_2_O_2_ in PBS for inhibiting endogenous peroxidase. EV71 was detected using polyclonal rabbit anti-EV71 antibody (a gift from Dr. Guan-Zeng Zheng, dilution, 1∶500). The antibody was prepared by vaccinating the rabbit with the whole inactive EV71 virus. The slides were incubated with peroxidase-conjugated anti-rabbit antibody (F9887, Sigma-Aldrich). Viral antigens in the tissue sections were visualized by peroxidase staining using DAB (Dako, Glostrup, Denmark) substrate followed by counterstaining with Mayer’s haematoxylin (Merck, Darmstadt, Germany). The control sections were incubated with medium instead of polyclonal rabbit anti-EV71 antibody.

### Quantitative Detection of EV71 RNA by RT-PCR

Tissues from gerbils were aseptically harvested in lysis buffer and were homogenized for total RNA using the RNeasy extraction kit (Qiagen, USA) according to the manufacturers’ instructions. Briefly, 600 µl of lysis buffer was added to 25 mg of homogenized tissue. The nucleic acids were eluted in 30 µl nuclease-free water and stored at −20°C. Viral load in different tissues was carried out using the TaqMan RT-PCR for the detection of EV71 RNA. Reactions were performed on a RT-PCR kit (Liveriver, ZJ Bio-Tech Co., Ltd. Shanghai). Each assay was carried out in triplicate. As described in previous report [23], the standard curve was developed with serial 10-fold dilutions of stock EV71 58301 strain (1×10^8.0^ TCID_50_/ml).

### Determination of Antibodies to EV71

Antibodies to EV71 were determined by indirect immunoﬂuorescence (IFA). EV71-infected Vero cells were dried onto 12 well slides, then fixed in acetone, and used for IFA. Sera from the EV71 inoculated gerbils were 2-fold serially diluted in PBS and a 10 µl volume was applied to each well of the slide and incubated for 30 min at 37°C. After washing with PBS for 10 min, the slides were labeled with 10 µl of fluorescein isothiocyanate (FITC)-labeled anti-mouse IgG (Sigma) at 37°C for 30 min. The slides were rinsed with PBS for 10 min, covered with cover slides, and examined under a fluorescent microscope (Leica, Germany).

### Vaccine Preparation

Confluent monolayers of Vero cells were inoculated with EV71 for 2 h, at 37°C, and then were replaced with MEM without fetal bovine serum. For the preparation of vaccine, a previously reported protocol was followed [24]. Briefly, EV71-infected Vero cell culture was incubated for 2–3 days till majority of the cells showed cytopathic effect (CPE), and the infected cells were then harvested followed by two freeze-thaw cycles to lyse the cells. The large cell debris was removed by centrifugation at 5000 g for 10 min at 4°C. Virus supernatant was loaded onto Sepharose 6 Fast Flow (GE Healthcare, USA) for further purification. After purification, the virus protein concentration was determined and virus was inactivated by mixing with 1∶4000(v/v) formaldehyde (Merck) at 37°C for 2 weeks followed by absorption with aluminum hydroxide (REHYDRAGEL LV, General Chemical, LLC) as adjuvant at a final concentration of 0.5 mg/ml.

### Vaccination and EV71 Challenge

Neonatal gerbils were injected IP with the EV71 vaccine (20 µg/gerbil, n = 8, per group) and boosted a week later. Another eight gerbils were used as controls. Two weeks after the second injection, the animals were given a lethal dose challenge (100×LD_50_). Gerbils were monitored daily for disease signs (hind limb paralysis) or death for 20 days.

### Data Analysis

The survival rates of gerbils infected with EV71 were analyzed by log-rank analysis. Clinical score curves were analyzed by Wilcoxon in GraphPad Prism, version 5.0(GraphPad 4 Software, San Diego, CA). Virus titers in tissues after EV71 infection are expressed as means±standard errors of the means (SEM) for 3 gerbils. A P≤0.05 was considered statistically significant.

## Results

### EV71 Infection in Gerbils

**Figure 1:**
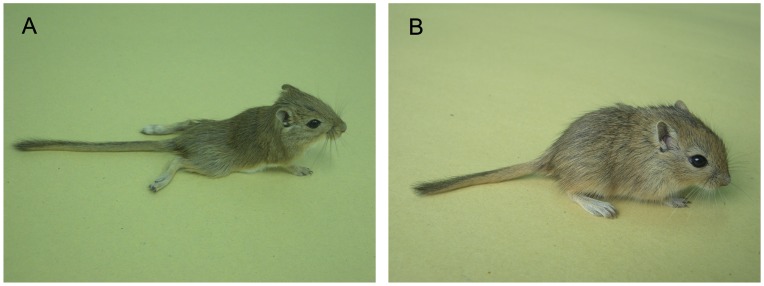
21-day-old gerbils infected with EV71 intraperitoneally (IP) at a dose of 1×10^4.0^ TCID_50_. (A) Photograph was representative picture of hind limb paralysis caused by 58301 at 6 dpi. (B) Photograph was representative picture of normal 21-day-old gerbils.

**Figure 2:**
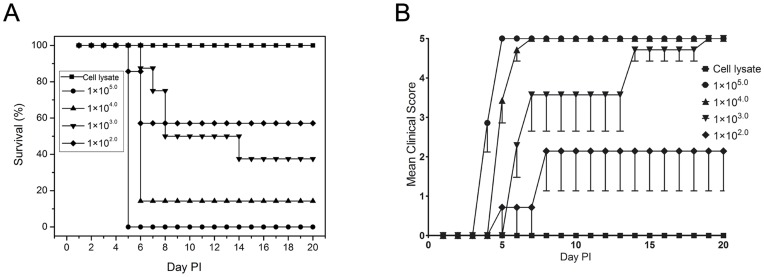
21-day-old gerbil infected IP with ten-fold serial dilution of EV71. Control animals were given uninfected Vero cell lysate. Survival (A) and clinical scores (B) were monitored daily after inoculation. Clinical scores were defined as following: 0,healthy; 1,ruffled hair, hunchbacked or reduced mobility; 2,limb weakness; 3,paralysis in 1 limb; 4,paralysis in both limbs; 5,death. Each group contained seven or eight gerbils. One representative of two independent experiments was shown.

In our previous studies we found that IP inoculation of 1×10^5.0^ TCID_50_ EV71 in gerbils aged between 7 and 28 days resulted in the death of the infected gerbils, and all the dead gerbils showed severe CNS-related pathological changes. In the gerbils aged from 28 days to 49 days, the gerbils in the younger age group sometimes died of the EV71 infection whereas gerbils in the older age group could develop hind limb paralysis induced by EV71 infection but no death. Gerbils older than 49 days showed no sign of disease, but majority developed antibodies against EV71 (data not shown). In order to further evaluate the EV71 infection model in detail, gerbils at the age of 21 days old were used in this study. Inoculation of gerbils IP with 1×10^5.0^ TCID_50_ EV71 resulted in death at 4–5 days postinfection (dpi). All gerbils (7/7) developed rapidly progressing weakness and lethargy and died within 6–8 h after the onset of symptoms. After inoculation with 1×10^4.0^ TCID_50_ EV71, 6/7(85.71%) gerbils had hind limb paralysis (Fig. 1) and gerbils died at 5 or 6 dpi. One surviving gerbil had sequelae of paralysis and showed EV71 seroconversion. The incidence of death was reduced to 62.5% (5/8) following inoculation with a dose of 1×10^3.0^ TCID_50_ and gerbils died at 7–14 dpi. Seven gerbils developed hind limb paralysis between 6 and 8 dpi. Two out of 3 surviving gerbils had detectable EV71 seroconversion. The surviving gerbils inoculated with 1×10^4.0^ or 1×10^3.0^ TCID_50_ EV71 showed the same changes in antibody levels. EV71 IgG was not detected until 7 dpi, and IgG titers gradually increase from 1∶160 (day 14) to 1∶640 (day 21) by the IFA assay. In the EV71-infected gerbils, we found a viral inoculum dose-dependence with respect to disease severity and death rate. For example, at the high dose, 1×10^5.0^ TCID_50_ EV71 induced death of all infected gerbils. At the dose of 1×10^4.0^ TCID_50_, all gerbils were infected, but one survived from the infection. At the low dose of 1×10^3.0^ TCID_50_, some gerbils were resistant to EV71 infection (no seroconversion**)**. LD_50_ value of EV71 infection via IP was 1×10^2.64^ TCID_50_ in 21-day-old gerbils (Fig. 2).

### Pathology in EV71-infected Gerbils

Using histopathological examinations we found typical lesions in various tissues in gerbils after IP injection with a dose of 1×10^5.0^ TCID_50_ EV71. Hind limb paralysis occurred at 4 or 5 dpi in all four gerbils. At this time point, the spinal cords and brainstem were the most severely affected organs in the CNS. The neuronal swelling, neuronophagia, necrosis of neuron and glial cell hyperplasia were observed in infected gerbils (Table 1).

**Figure 3:**
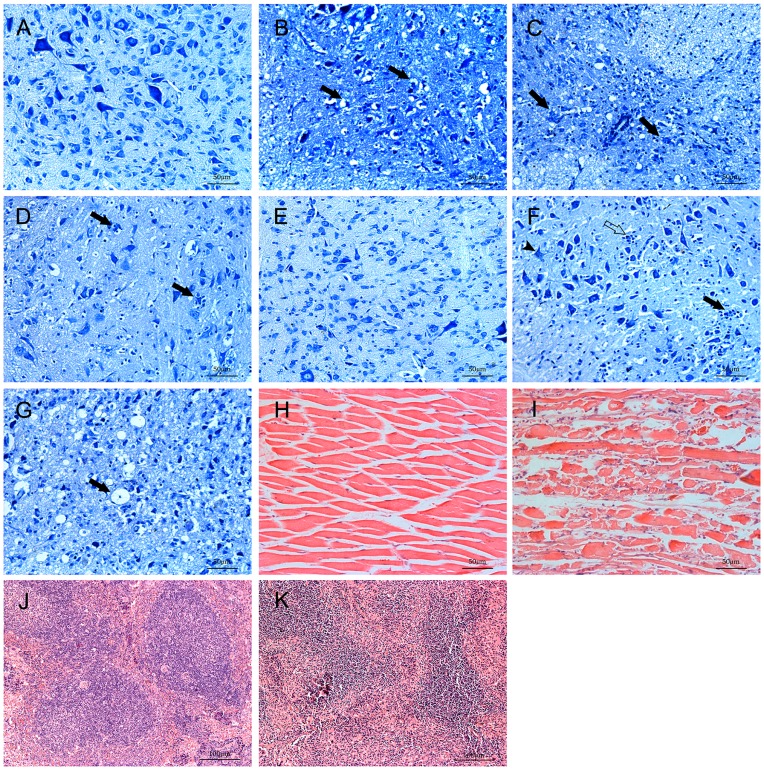
Pathologic changes in various tissues of EV71-infected gerbils. 21-day-old gerbils were inoculated with EV71 (1×10^5.0^ TCID/gerbil) IP or Vero cell lysate (mock control) and tissues were examined histopathologically at 4–5 dpi. Pathologic lesions could be seen in infected gerbils. (B) Spinal cord presented as a sieve-like change and nerve cells arranged disorder and some were swelling (solid arrow) with decrease of Nissl bodies. (C)Nerve cells became disintegrated, necrotic and disappearance of the nucleus (solid arrows). (D)Multiple foci of microglial accompanied by neuronophagia (solid arrows) were in anterior horn cells. (F)In brainstem, neuronophagia (solid arrows), neuronal swelling (solid arrow-heads) and microglial (open arrows) were seen. (G)The myelin sheath expansion (solid arrows) was occasionally observed. Severe necrotizing myositis in the skeletal muscle (I) and splenic atrophy (K). Normal tissues were shown (A, E, H and J). (A–G) Nissl stain, (H–K) Hematoxylin and eosin stain.

**Figure 4:**
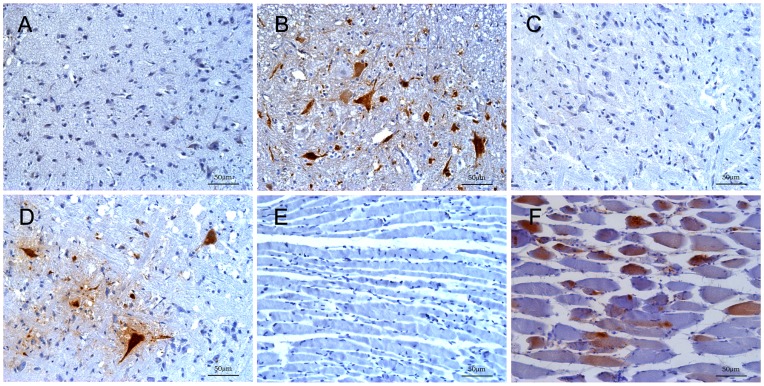
Immunohistochemistry of tissues in EV71- infected gerbils. 21-day-old gerbils were inoculated IP with 1×10^5.0^ TCID/gerbil of EV71 at 4–5 dpi. The neuron and adjacent sections were positive for EV71 antigen in the spinal cord which was mainly located in gray matter (B). The neuron was viral antigen-positive in the brainstem (D). Viral antigen was detected in the skeletal muscle fibers (F). Mock controls were shown (A, C and E). Immunohistochemistry with DAB chromogen and counterstain with hematoxylin.

There were a sieve-like change, nerve cells arranged disorder and swelling with decrease of Nissl bodies in the spinal cord of infected gerbils (Fig. 3B). Nerve cells became disintegrated and necrotic indicated by cytoplasmic vacuolization, pyknotic nuclei and disappearance of the nucleus, which resulted in significant reduction of nerve cells (Fig. 3C). Multiple foci of microglial accompanied by neuronophagia can be prominently observed in anterior horn cells in spinal cord (Fig. 3D). In brainstem, neuronophagia, neuronal swelling and microglial were also seen (Fig. 3F). In addition, the myelin sheath expansion were occasionally observed (Fig. 3G), where virus replication could often be detected in CNS (Table 1). Meanwhile, the EV71 antigen could be detected in these tissues by immunohistochemistry (Fig. 4B and 4D). No obvious neuronal abnormalities were identified in the brain cortex. Occurrence of different types of pathological lesions was summarized in Table 2. Skeletal muscle edema, severe necrotizing myositis with lymphocytic inflammatory infiltration and interstitial edema were observed in the limb muscles (Fig. 3I). We also found the periarterial lymphatic sheaths became thinner, severe myeloid and lymphoid depletion in the spleen of infected gerbils (Fig. 3K). In contrast, tissue samples collected from uninfected gerbils showed no pathological changes (Fig. 3A, 3E, 3H and 3J). In each infected gerbils, the spinal cord, brainstem and muscle appeared to be the organs where the most severe lesions were detected. Furthermore, the EV71 antigen could frequently be detected in these organs with anti-EV71 antibody strongly indicate the lesions were induced by EV71 infection. The spinal cord, brainstem and limb muscle showed an intensive staining of EV71 antigens, indicating an active virus replication in these tissues (Fig. 4B, 4D and 4F), while organs such as liver, intestine, spleen and brain cortex were negative for EV71 antigens. Although obvious inflammatory cell infiltration can be seen in the lung (detailed results will be reported in a separate manuscript), no visible EV71 antigen was detected by immunohistochemistry.

**Table 1:** Clinical signs, pathological changes and virus replication in EV71-infected gerbils.

Gerbils	Clinical signs	Days post infection	Pathological findings in spinal cords or brainstems	Virus titer (log_10_ TCID_50_/g)		
				Spinal cords	Brainstems	Skeletal muscles
1	Hind limb paralysis, minimal movement, lethargy	4	Neuron swelling, neuronophagia, necrosis of neuron, glial cell hyperplasia	8.5	7.6	8.5
2	Hind limb paralysis, somemovement possible, tachypnea	5	Neuron swelling, necrosis of neuron, neuronophagia, axonal degeneration	7.9	7.8	8.1
3	Hunched posture, hind limbparalysis, minimal movement	5	Neuron swelling, neuronophagia, glial cell hyperplasia, necrosis of neuron	8.3	8.1	7.8
4	Hind limb paralysis, somemovement possible	5	Neuron swelling, glial cell hyperplasia	8.2	7.5	8.2

Four 21-day-old gerbils were inoculated IP with a dose of 1×10^5.0^ TCID_50_ EV71.

**Table 2:** The pathological types of CNS in EV71-infected gerbils.

Pathological findings	Spinal cords (Total = 4)		Brainstems (Total = 4)	
	No. of animals	%	No. of animals	%
Neuron swelling	4	100	3	75
Neuronophagia	3	75	3	75
Necrosis of neuron	3	75	3	75
Glial cell hyperplasia	3	75	2	50
Axonal degeneration	0	0	1	25

Four 21-day-old gerbils were inoculated IP with a dose of 1×10^5.0^ TCID_50_ EV71.

### EV71 Replication in Tissues of Infected Gerbils

**Figure 5:**
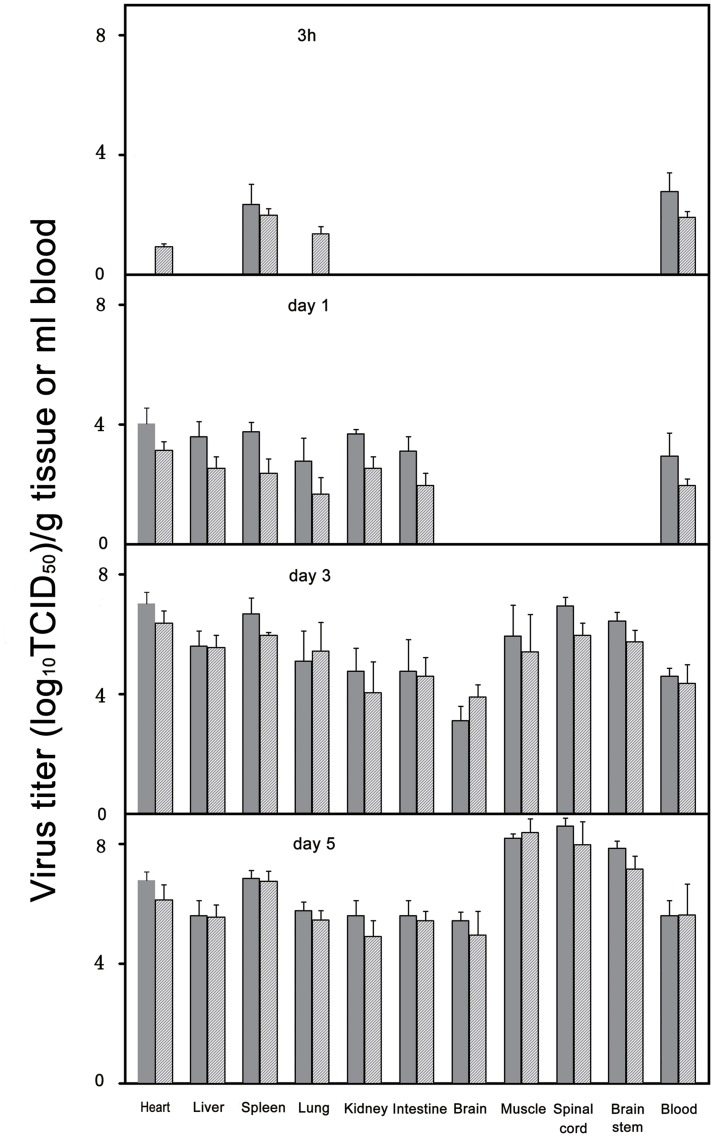
Virus replication in various tissues of EV71-infected gerbils. Twelve 21-day-old gerbils were inoculated EV71 IP with a dose of 1×10^5.0^TCID_50_ and tissue samples were collected at the time points indicated (3 gerbils/each). Virus titer in culture (solid bars) was expressed as TCID_50_ per milliliter (blood) or per gram (tissue). Calculated virus titer (hatched bars) was based on standard curve of real-time RT-PCR obtained with 10-fold serial dilutions of virus strain 58301. The data shown here are the mean virus titers ±standard errors (n = 3 each). Results are representatives of two independent experiments. The data between the virus titer in culture and calculated virus titer were no statistically significant by student’s t-test.

In order to further understand the target organs and tissues of EV71 in infected gerbils, we collected tissue samples from EV71-infected gerbils including spinal cord, heart, brainstem, brain cortex, muscle, liver, lung, kidney and blood. Virus isolation and titration were performed at different time points (3 h, 1 day, 3 days and 5 days) after the gerbils were inoculated with 1×10^5.0^ TCID_50_ EV71 using Vero cell culture. At 3 h post-infection, EV71 can only be detected in the spleen and blood with relative low viral titers (1×10^2.0^ to 1×10^3.0^ TCID_50_/g) and the viral titers gradually increased (1×10^4.0^ to 1×10^6.0^ TCID_50_/g) at 3 dpi in all tissues (Fig. 5), demonstrating the eclipse phase and suggesting viremic spread of EV71. The virus was found in tissue samples from lung, heart, liver, kidney and intestinal with a viral titers from 1×10^3.0^ to 1×10^4.0^ TCID_50_/g at 1 dpi. Interestingly, no virus could be detected in the muscles until 2 dpi. A high virus titer (1×10^7.0^ TCID_50_/g) was detected in the brainstem and spinal cord at 3 dpi and increased gradually in virus titer to 1×10^8.5^ TCID_50_/g at 5 dpi, indicating that EV71 was transmitted to the CNS from the viremia. In general, virus titers were medium high (1×10^6.0^ TCID_50_/g) in the liver, lung, kidney, blood and intestine at 5 dpi. Much higher virus titers were found in the spinal cord, brainstem and skeletal muscle, which could reach 1×10^7.5^–1×10^8.5^ TCID_50_/g. The spinal cord, brainstem and skeletal muscle maintained high virus titers indicated that these tissues were sites of ongoing virus replication.

### Protection Against EV71 Challenge in Vaccinated Gerbils

**Figure 6:**
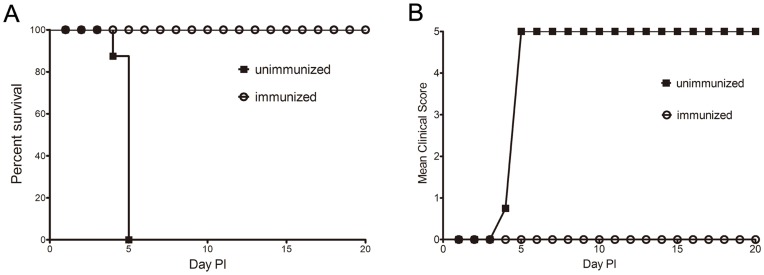
Survival and clinical score of immunized gerbils after IP challenge with lethal doses of EV71. Experiment groups (n = 8) were immunized with the EV71 inactive whole-virus vaccine at birth and boosted a week later. The control groups (n = 8) were immunized with normal saline. All gerbils were challenged by the IP route with lethal doses (100×LD_50_) of EV71 at day 21. The survival rates and clinical scores were monitored daily after challenge. Clinical scores were graded as described in the legend of Figure 2.

To evaluate whether the gerbil model can be used for testing protective immunity induced by vaccination with inactive whole-virus vaccine, neonatal gerbils were immunized with the inactive EV71 vaccine at birth followed by a booster dose at day 7. Gerbils (n = 8) receiving normal saline served as negative controls [24]. Both vaccinated and control gerbils were challenged by EV71 IP at the dose of 100×LD_50_ on day 21. The experimental gerbils were observed for paralysis or death for the next 20 days. In vaccinated groups, 100% of gerbils were protected from EV71 challenge showing no weight loss, paralysis or death. In contrast, the control gerbils developed progressive hind limb paralysis and died on days 5 after the EV71 challenge (Fig. 6). Furthermore, no virus could be isolated from the muscle of the vaccinated gerbils. The result strongly indicates whole-virus vaccine completely protected gerbils from EV71 infection and the gerbil model can be used for evaluation of protective immune responses induced by EV71 vaccination.

## Discussion

Previously reported studies in rodent models revealed only newborn mice were shown to be susceptible to EV71 infection with clinical symptoms but no damage to CNS, and neuronal lesions could only be induced by EV71 strains that have been adapted by serial passages in mouse brains. When mice reach 6 days of age, they are no longer susceptible to EV71 infection. For EV71 freshly isolated from patients, only neonatal mice are susceptible [18], [19], [25]. Therefore, these models can not be conveniently utilized as a disease model for either vaccine evaluation or studies of virus-mediated pathogenesis.

In this study, we investigated whether gerbils could be used as a sensitive model for EV71 infection. Gerbil is a small mammal of the order Rodentia, which have been used as a reliable animal model for a variety of parasitic, bacteria and virus infections, including filariasis, helicobacter pylori and hemorrhagic fever with renal syndrome [26], [27], [28]. By IP injection of freshly isolated EV71 in gerbils aged 21-days, we found that infection of this non mouse-adapted EV71 resulted in disease symptoms related to CNS damages. This result also had been repeated by other clinical isolated EV71 strains (data not shown). Interestingly, the CNS symptoms and death rates were correlated well with infection dose, showing more severe symptoms and higher death rate in the high dose group than in the low dose group (p<0.05). Although EV71 can be detected in many types of tissue from dead gerbils, the virus titers are much higher in spinal cord, brainstem and skeletal muscles than other types of tissues or organs, strongly indicating the CNS is the major target organ for EV71 infection. Histopathologic changes could be observed in CNS such as the spinal cord, brainstem, and brain cortex, as well as non CNS organs such as skeletal muscles and spleen. The neuropathologic changes included neuronal degeneration, neuronal loss, necrosis and neuronophagia in the CNS. These neurological lesions are very similar to those described in EV71 patients with CNS symptoms [29].

The virulence of a given mouse-adapted EV71strain is higher than its original freshly isolated strain after serial passages in mouse brain. For example, infection of a mouse-adapted stain in 1-day-old ICR mice results in the death much quicker (2 to 3 dpi) than infection with a similar dose of the original virus (8 to 9 dpi). LD_50_ in mice descended from 5.0×10^4.0^ p.f.u. per mouse for the freshly isolated strain to 4.2×10^2.0^ p.f.u. for the adapted strain [18]. In this gerbil model, we demonstrated that EV71 infection could induce death in 21-day-old gerbils with a fresh clinical isolate at a dose of 1×10^5.0^TCID_50_. The LD_50_ value was 1×10^2.64^ TCID_50_, which was lower than that for the adapted strain in neonatal ICR mice. This result indicates that gerbils are more susceptible to EV71 infection than newborn ICR mice. In addition, in the newborn ICR mouse model, no obvious tissue damage and inflammation can be detected in CNS by infecting with a freshly isolated virus strain suggesting that EV71 infection in ICR mouse model behaves more like an infection with a myotropic virus. As reported, in the ICR mouse model, only mouse-adapted strains were neurotropic [14], [16], [30]. In contrast, the gerbil infected with the non mouse-adapted EV71 strain exhibited CNS-related lesions. We believe the gerbil model may serve as a valuable disease model for studying pathologic mechanisms of EV71 infection compared with the current ICR model. Moreover, since gerbils aged 21 days are still very susceptible to freshly isolated EV71 strains and show similar neurologic lesions in patients, the model also shows the potential value for predicting and evaluating the virulence of the virus strains directly isolated from patients.

Developing effective vaccines is considered the top priority for preventing EV71 infection among all other approaches [31]. Several types of vaccine candidates have been developed against EV71infection including heat-inactivated or formaldehyde-inactivated whole-virus [14], [24], EV71 virus-like particles (VLP) [32], [33], recombinant VP1 protein, and DNA vaccines [31], [34]. Systematic evaluation of protective efficacy and safety of these vaccine candidates requires a reliable animal model. Since mice aged 7 days or older were resistant to EV71 challenge, EV71 vaccine candidates have usually been evaluated by immunizing pregnant mice and challenging their pups [14], [19], [30], [31]. Alternatively, mouse-adapted EV71 has been used for challenge to test the protective efficacy of vaccines [24]. Obviously, the disadvantage of the first model is that it only evaluates efficacy of passively immunization from the mother mice to suckling mice. The disadvantage of the second model is that it requires a mouse-adapted strain, so may not be relevant for vaccines designed for human strains and does not have the flexibility to be used for heterologous viral challenges. Although neutralizing antibodies induced by vaccine could indirectly evaluate protective immunity, high titer neutralizing antibodies are not universally associated with protection [14]. It is apparent that potent protective activity against the viral infection in animal models has been considered as the best system for pre-clinical vaccine evaluation. [30]. In this report, we found that 21-day-old gerbils are very sensitive to EV71 strains directly isolated from a clinical sample, which obviates the need of using mouse-adapted virus strains. Furthermore, being able to challenge at day 21 or later allows a candidate vaccine regimen to induce and immune response that can be directly evaluated by challenging with EV71 strains, which apparently can not be achieved using the current newborn ICR mouse model. The gerbil model described here provides a way to test the protective efficacy of active vaccination against lethal challenge with a variety of EV71 strains directly from patient samples, which will be more reliable than the approach of using mouse-adapted strains.

In summary, a disease model for EV71 infection was established in young gerbils at the age of 21 days. The young gerbils are susceptible to non mouse-adapted EV71 infection and shows clinical symptoms similar to those in patients. Most importantly, in the gerbils, EV71 susceptible phase was extended to the age of 21 days, allowing a big testing window time for accurately assessing protective immunity generated by EV71 vaccination, and therefore, the gerbil model showed significant advantages over other animal models such as the newborn ICR mouse model for EV71 vaccine evaluation. Moreover, the EV71-induced neurological disorders in young gerbils may serve as a valuable disease model for understanding EV71 pathogenesis.
